# Invasive carcinoma in accessory axillary breast tissue: A case report

**DOI:** 10.1016/j.ijscr.2019.05.037

**Published:** 2019-05-28

**Authors:** Ruqayya Naheed Khan, Muhammad Asad Parvaiz, Amina Iqbal Khan, Asif Loya

**Affiliations:** a FCPS, SKMCH, Pakistan; b SKMCH, Pakistan

**Keywords:** Accessory breast tissue, Breast carcinoma, Ectopic breast tissue

## Abstract

**Introduction:**

Accessory or ectopic breast tissue is an aberration of normal breast development. It is known to be a rare entity present anywhere along the embryologic mammary streak or milk line but more common in axilla.

**Presentation of case:**

We report a case of 36 year old female with accessory breast carcinoma who presented with a progressive lump in her left axilla for 1 year. On examination a 2 cm solitary mass was palpable in axilla. Ultrasound confirmed a 19 mm mass with no other lesion in breast and axilla. Core biopsy showed invasive ductal carcinoma. She was discussed in multidisciplinary board meeting and was offered upfront surgery with excision of accessory breast tissue and sentinel lymph node biopsy. Axillary lymph node dissection was omitted following ACOSG Z0011 criteria. She was offered adjuvant chemotherapy and radiation post operatively along with endocrine treatment as she was hormone receptor positive.

**Discussion:**

Accessory breast development is hormone dependent just like normal breast. Breast cancer in accessory breast tissue is very rare. The incidence is around 6%. Most common pathology is invasive ductal carcinoma (50–75%). The most common location is axilla (60–70%) although it can present in other less common locations like infra-mammary region (5–10%) and rarely in thighs, perineum, groin, and vulva.

**Conclusion:**

Since accessory axillary breast tissue is out of the image of screening breast examination, it is necessary for the oncologists to be aware of this entity and associated pathologies. Their preventive excision in high risk women can also be considered.

## Highlights

• Accessory breast cancer is a rare entity with incidence of around 0.2%–0.6%.
• Routine mammograms can miss accessory breast tissue due to its location.
• General lack of awareness of accessory breast cancer among clinicians has potentially dangerous implications.
• Accessory breast cancer does not require mastectomy unless involved by cancer.

## 1 Background

Polymastia is a congenital condition in which abnormal accessory breast tissue is found in addition to normal breast. Accessory breast tissue is an aberration of normal breast with reported incidence of 0.3–6% [1,2] in general population and more common among Asians and Caucasians [3]. Their development and pathologies are same as normal breast. Patients are usually unaware of their presence unless it becomes pathologically involved in the form of inflammation, benign lumps and malignancy. The incidence of cancer originating from this is around 0.2%–0.6% [4]. The outcome of accessory breast cancer is known to be poor due to its rarity, early lymph nodes involvement and late diagnosis [4]. In this report we present a case of accessory axillary breast carcinoma in a young female. The work has been reported in line with the SCARE criteria [5].

## 2 Case report

### 2.1 Clinical course

**Fig. 1:**
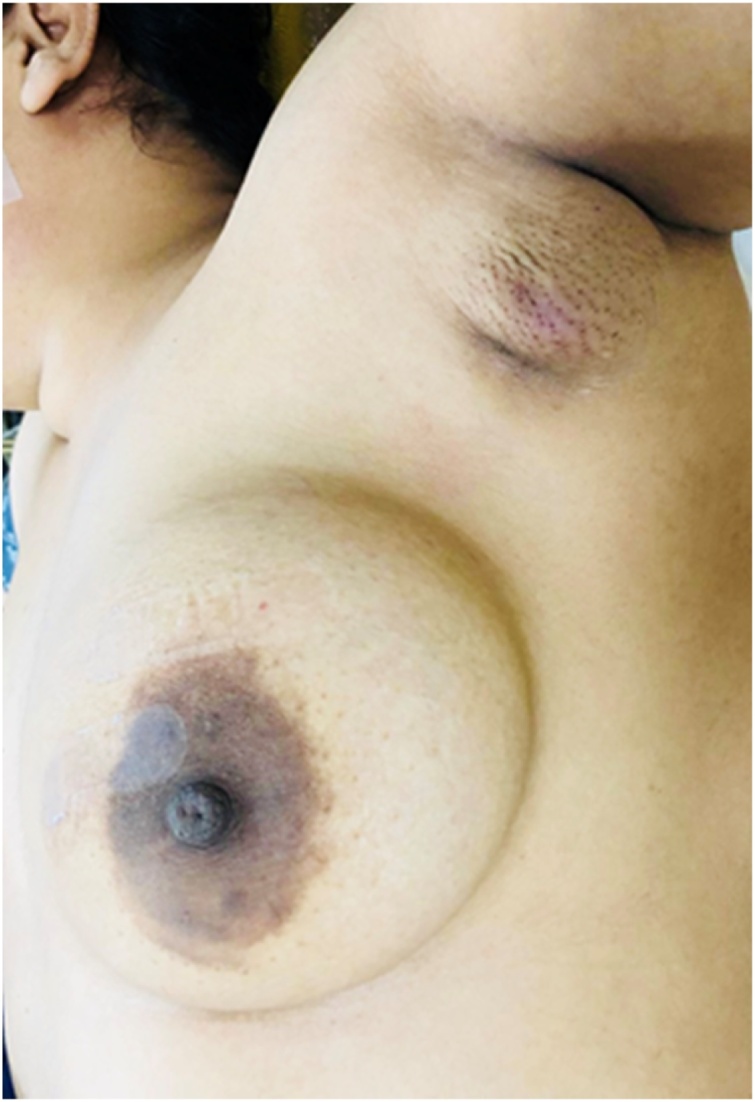
Clinical Photograph.

A 36 year old premenopausal multiparous woman presented to us in one stop breast clinic with a lump in her left axilla for last one year. It was slowly increasing in size with no other associated symptoms. She had no medical comorbidities or family history of breast cancer. On examination a 2 cm irregular mass with skin tethering was palpable in left axilla with the presence of bilateral axillary breast tissue. No lump was palpable in both breast and no axillary lymphadenopathy at the time of presentation. Systemic examination was unremarkable (Fig. 1).

### 2.2 Investigations

**Fig. 2:**
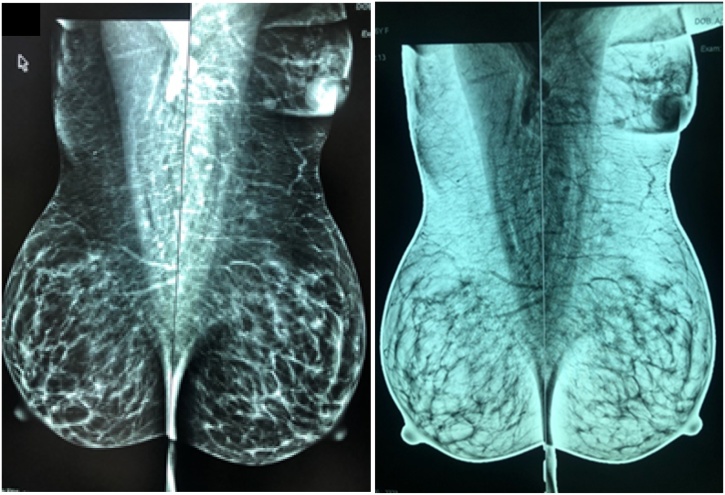
Mediolateral View Mammogram.

Routine bilateral breast ultrasound and mammogram was done which confirmed the presence of a 1.5 × 1.8 × 1.9 cm mass in the left axilla. No solid or cystic mass was identified in both breasts. No suspicious lymph nodes were seen. Magnetic resonance imaging confirmed above findings (Fig. 2).

A trucut biopsy from the lump was performed which revealed invasive ductal carcinoma grade II with no lymphoid tissue in the background. Further routine immunohistochemical analyses were performed on formalin fixed, paraffin embedded specimens which included ER, PR, HER2Neu and Ki67. Estrogen receptors were positive in 20% of cells, progesterone receptors were positive in 25% of cells, HER2Neu was positive with score of 3+ while Ki67 proliferative index was 40%.

Chest x-ray, abdominal ultrasound and bone scan were normal. A diagnosis of left accessory breast cancer with clinical stage I (T1N0M0) was made.

### 2.3 Treatment strategy

**Fig. 3:**
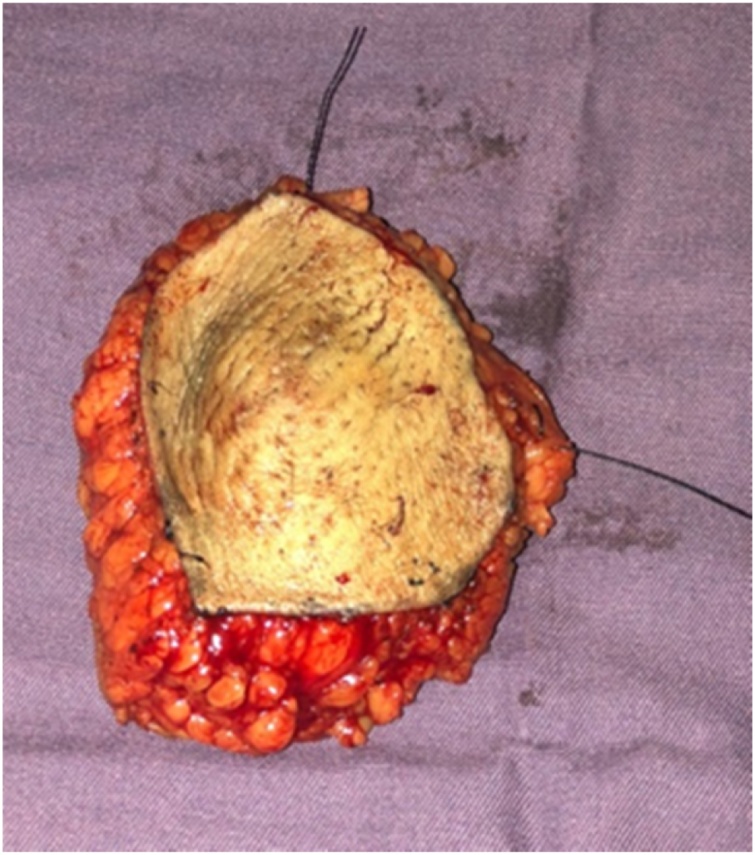
Specimen Photograph.

Patient was discussed in multidisciplinary team meeting and was planned for an upfront wide local excision of entire accessory breast and sentinel lymph node biopsy. Wide local excision with an ellipse of extra skin was done. Sentinel lymph node biopsy was performed using gamma probe. 3 sentinel lymph nodes were identified and sent for intra-operative frozen Section. 2 out of 3 lymph nodes were reported as positive for metastasis by 2 pathologists. Size of metastatic deposit was 12 mm and 1 mm. There were no other suspicious looking lymph nodes in axilla. Further axillary lymph node dissection was omitted. We routinely manage axilla according to ACOSG Z0011 protocol at our hospital where axillary lymph node dissection is spared in 1–2 positive lymph nodes. Axillary dissection is done with 3 or more positive lymph nodes (Fig. 3).

Histopathological examination of the specimen revealed a 28 mm invasive ductal carcinoma grade III. Tumor was 2 mm away from nearest lateral margin with no ink on tumor margin. Skin was free of tumor. Final histopathology of sentinel lymph nodes confirmed intra-operative frozen section report. Pathologically it was staged as pT2N1M0 according to pTNM, AJCC 8^th^ edition.

She was offered 4 cycles of Adriamycin-cyclophosphamide, 4 cycles of Taxol with Herceptin followed by radiotherapy to ipsilateral breast and axilla according to the standard protocol. She was put on hormonal treatment later on. Patient is on routine 6 monthly follow up. Her yearly mammogram and clinical examination have been negative for any recurrence for past 2 years.

## 3 Discussion

Bilateral embryological mammary streaks or milk line are thickened strips of ectoderm running along the ventral surface of the body from anterior axillary fold to medial aspect of inguinal fold symmetrically on both sides. They involute during embryogenesis except in thoracic region to give rise to breast tissue [6]. Their persistence anywhere along the line can result in accessory breast. This accessory breast may just contain glandular tissue only or in combination with nipple areola complex. Their development is hormone dependent just like normal breast and usually become apparent during puberty and pregnancy. Since they behave just like normal breast, their pathologies are similar to normal breast which include pain, inflammation, fibroadenoma and carcinoma. As described earlier the incidence of carcinoma in accessory breast tissue is around 6% [4]. Most common pathology is invasive ductal carcinoma (50–75%) but there have been scattered case reports of medullary, lobular and phylloides as well [8,9]. The most common location is axilla (60–70%) although it can present in other less common locations like infra-mammary region (5–10%) and rarely in thighs, perineum, groin, vulva [7,8].

Differentials for axillary masses include axillary lymphadenopathy, lipoma, abscess, seroma, hidradenitis, fibroadenoma and cutaneous lesions. Treatment for cancer is usually delayed due to wide variety of differentials and lack of awareness regarding accessary breast cancer.

Accessory breast cancer diagnosis and management is done under same principles as breast cancer since there is not much data available in the literature due to its rarity to ascertain a different approach. This is one of the reasons in delayed administration of appropriate treatment to such patients as well as lack of awareness among clinicians regarding its existence.

Detail history, clinical examination along with a trucut biopsy is performed whenever a suspicious mass is palpated in axilla or along milk line. Routine mammograms can miss accessory breast tissue due to its location. An ultrasound is performed in addition to mammography. Metastatic workup is also done once diagnosis has been confirmed on core biopsy which includes bone scan and a CT-scan to exclude distant metastasis as well as a second primary other then breast. It is managed by surgery post chemotherapy or pre chemotherapy according to tumor size followed by radiation and endocrine treatment if applicable.

Surgical management has been a little controversial. Some authors like Matsuoka recommend mastectomy of ipsilateral breast if axilla is found involved by the carcinoma [[10], [11], [12]]. While others like Cogwells and Czerny have reported that ipsilateral mastectomy in accessory breast cancer patients does not result in a better prognosis [13]. Evans and Guyton concluded that there was no additional advantage of mastectomy versus local excision of accessory breast along with axillary lymph node dissection [14]. Mastectomy can be considered only in those patients who have an additional lesion in breast. However if clinical examination and investigations like mammogram and MRI exclude its presence then patient can be spared of mastectomy with a close follow up.

Axillary lymph node dissection is done only if lymph nodes are positive for metastasis. However it can be avoided in patients who fit in ACOSG Z0011 criteria since it is safe to omit axillary lymph node dissection in 1–2 positive lymph nodes [15,16].

Systemic adjuvant or neo-adjuvant chemotherapy is administered on same principles in accessory breast cancer as for anatomic breast cancer. Anthracyclines, such as doxorubicin or epirubicin, decrease the annual cancer recurrence risk by 2% while taxanes such as paclitaxel and docetaxel are suitable for patients with positive lymph nodes [17]. Radiotherapy of the tumor site must be done if axillary lymph node dissection has been avoided due to low burden disease however radiotherapy to the ipsilateral anatomic breast is controversial [18].

It is difficult to assess the prognosis of accessory breast cancer due to late presentation and lack of follow up data. A retrospective review of 11 accessory breast cancer patients was done by Shuo Zhang in 2015. All the patients were between the ages of 27yrs–48yrs with a median follow up of 20 months. Axillary lymph node dissection was performed in 6 patients. 4 patients presented with distal metastasis. The three years overall survival rate was 54.5% (6/11) due to late presentation with metastasis [19].

## 4 Conclusion

There is general lack of awareness among clinicians due to under reporting of accessory breast lesions which has potentially dangerous implications due to underestimation. Although carcinoma arising in accessory breast tissue is a rare diagnosis, its possibility should be considered. Accessory axillary breast tissue is out of the image of screening breast examination; it is necessary for the oncologists to be aware of this entity and associated pathologies. They should always be looked for while examining a patient for breast cancer and any changes should be noted. Their preventive excision in high risk women can also be considered.

## Conflicts of interest

The authors declare no conflict of interest.

## Funding

This manuscript has received no funding.

## Ethical approval

The case report has been exempted by the ethical committee of our institution (EXMPT-26-12-18-01).

## Consent

“Written informed consent was obtained from the patient for publication of this case report and accompanying images. A copy of the written consent is available for review by the Editor-in-Chief of this journal on request”.

## Author contribution

Ruqayya Naheed Khan: Paper writing.

Muhammad Asad Parvaiz: Primary surgeon/Conception of work.

Amina Iqbal Khan: Critical revision of article.

Asif Loya: Pathologist/Final approval of the version to be published.

## Registration of research studies

N/A.

## Guarantor

Muhammad Asad Parvaiz.

Amina Iqbal Khan.

## Provenance and peer review

Not commissioned, externally peer-reviewed.
